# Altering second-order configurations reduces the adaptation effects on early face-sensitive event-related potential components

**DOI:** 10.3389/fnhum.2014.00426

**Published:** 2014-06-12

**Authors:** Pál Vakli, Kornél Németh, Márta Zimmer, Stefan R. Schweinberger, Gyula Kovács

**Affiliations:** ^1^Department of Cognitive Science, Budapest University of Technology and EconomicsBudapest, Hungary; ^2^Institute of Psychology, Friedrich Schiller University of JenaJena, Germany; ^3^DFG Research Unit Person Perception, Friedrich Schiller University of JenaJena, Germany

**Keywords:** second-order relations, face processing, N170, neural adaptation, face aftereffect

## Abstract

The spatial distances among the features of a face are commonly referred to as second-order relations, and the coding of these properties is often regarded as a cornerstone in face recognition. Previous studies have provided mixed results regarding whether the N170, a face-sensitive component of the event-related potential, is sensitive to second-order relations. Here we investigated this issue in a gender discrimination paradigm following long-term (5 s) adaptation to normal or vertically stretched male and female faces, considering that the latter manipulation substantially alters the position of the inner facial features. Gender-ambiguous faces were more likely judged to be female following adaptation to a male face and vice versa. This aftereffect was smaller but statistically significant after being adapted to vertically stretched when compared to unstretched adapters. Event-related potential recordings revealed that adaptation effects measured on the amplitude of the N170 show strong modulations by the second-order relations of the adapter: reduced N170 amplitude was observed, however, this reduction was smaller in magnitude after being adapted to stretched when compared to unstretched faces. These findings suggest early face-processing, as reflected in the N170 component, proceeds by extracting the spatial relations of inner facial features.

## INTRODUCTION

Human faces invariably contain the same basic features positioned in the same fashion. This basic feature configuration is called first-order relational information (CONF_1st_; Diamond and Carey, 1986) and distinguishes the category of faces from other non-face object categories (Maurer et al., 2002). The variations of metric distances between these facial features is referred to as second-order relational information (CONF_2nd_; Diamond and Carey, 1986). Results show that humans are highly sensitive to such CONF_2nd_ (Haig, 1984) and it has been suggested that they are important for face recognition and the discrimination of individual faces from each other (Tanaka and Farah, 1991; Tanaka and Sengco, 1997; Leder and Bruce, 2000; Rotshtein et al., 2007; Richler et al., 2009).

Although previous results underline the importance of CONF_2nd_ in the representation of face identity, this view has been challenged more recently. First, it has been shown that face recognition based exclusively on these properties is relatively poor when they remain within the range of real-world variations (Taschereau-Dumouchel et al., 2010). Second, geometrical distortions that affect second-order relations have little or no effect on face recognition performance either (Hole et al., 2002), suggesting that the extraction of simple distances between facial features is not crucial for face recognition.

In the past few years, electrophysiological studies have focused on the N170 event-related potential (ERP) component or on its magneto-encephalographic counterpart, the M170, which are face-specific in the sense that they are usually larger to faces than to non-face objects (Bentin et al., 1996; Itier and Taylor, 2004; Gao et al., 2013; Rivolta et al., 2014; for review see Eimer, 2011; Rossion and Jacques, 2011). It has been suggested that the N170 is sensitive to the CONF_1st_ of faces. For example, presenting the same facial features in a scrambled configuration reduces the amplitude of the N/M170 (e.g., Bentin et al., 1996; Gao et al., 2013) while stimulus inversion, that interrupts configural face processing (Yin, 1969), delays and enhances N170 as compared to upright faces (Eimer, 2000a; Rossion et al., 2000; Wiese et al., 2009). Therefore it seems that the N/M170 electromagnetic component is associated with the early and generic structural processing of faces, related to the category of faces per se (Bentin et al., 1996; Jeffreys, 1996; Schendan et al., 1998; Eimer, 2000a,b; Joyce and Rossion, 2005; Kloth et al., 2010; Ganis et al., 2012; Gao et al., 2013).

One aspect, however, that remained largely neglected is the relation of the N/M170 to the processing of CONF_2nd_. Some results suggest that the N170 is relatively insensitive to manipulations that change the CONF_2nd_. In a previous study using a passive viewing paradigm, altering faces by displacing the eyes and mouth and hence changing the CONF_2nd_ while leaving the CONF_1st_ intact did not modulate the amplitude or the latency of the N170 component (Halit et al., 2000). The N170 was, however, larger in amplitude in response to faces that were judged atypical and unattractive when compared to typical and attractive ones. The authors concluded that the N170 may be related to the encoding of faces in relation to a general face prototype, whereas individual recognition mechanisms may be reflected in the later P2 component which indeed showed sensitivity to the configural modification of faces (Halit et al., 2000). In a more recent experiment, participants were presented with pairs faces that differed either in their local features or their CONF_2nd_ properties (Mercure et al., 2008). The N170 did not show any difference between featurally or configurally manipulated faces neither when the participants had to make same/different judgements, nor when they were explicitly instructed to focus on the featural or configural differences between the members of each face pair. On the other hand, other studies suggest that the N170 of the right hemisphere reflects neural functions that are related to the processing of CONF_2nd_ as well (Scott and Nelson, 2006; Zimmer and Kovacs, 2011). Scott and Nelson (2006) recorded ERPs to previously familiarized faces in which either the eyes and mouth were displaced while leaving the CONF_1st_ unaffected, or the same features were replaced by those of another individual without any change in their position. In a passive viewing paradigm, the overall amplitude and latency of the N170 did not differ in response to the original familiar and modified face stimuli. On the other hand, when analyzing difference waveforms (obtained by subtracting the ERP responses for the altered faces from those evoked by the original ones), the authors found a greater N170 amplitude difference for configural than for feature changes over the right hemisphere. The opposite pattern was observed over the left hemisphere. This result is indicative of the role of CONF_2nd_ in the processing of faces as reflected in the N170 component. Moreover, it has also been demonstrated that adaptation of the N170, that is, the reduction of its amplitude to face repetition is evident and even enhanced over the right hemisphere for faces with expanded and contracted inner features (Zimmer and Kovacs, 2011).

Taken together, the few studies mentioned above yield mixed results regarding whether the N170 reflects face processing mechanisms engaged in the coding of CONF_2nd_ of faces. Another stimulus manipulation that changes the aspect ratio and hence the CONF_2nd_ of faces without affecting CONF_1st_ is stretching the entire face along one of its axes (Hole, 2011). It has been shown that human face recognition is surprisingly robust to stretching (Hole et al., 2002; Bindemann et al., 2008). In a repetition-priming paradigm Bindemann et al. (2008) found that the presentation of stretched and normally proportioned primes leads to no repetition-related effects for the N170 at all, and repetition effects in the subsequent N250r component were equivalent for both prime conditions. However, recent results suggest that exclusive neural mechanisms underlie priming and adaptation-aftereffects (Walther et al., 2013). More specifically, Walther et al. (2013) have shown that behavioral priming (reduced response times and increased accuracy in identity classification for repeated faces) and aftereffects (contrastive perceptual biases in identity judgment) can be demonstrated within a single paradigm for unambiguous and ambiguous faces, respectively. Importantly, the two effects never occurred concurrently for the same stimuli, indicating that distinct mechanisms can account for these phenomena. Therefore it is possible that the paradigm of Bindemann et al. (2008) is less suited to test the earlier structural encoding steps of face processing reflected in the N170. In the current experiment we applied an adaptation paradigm (Webster and MacLin, 1999) involving face gender judgments that has previously been shown to lead to robust reductions of the N/M170 (Kovacs et al., 2006; Harris and Nakayama, 2008; Kloth et al., 2010) to test whether changing the aspect ratio of faces changes the adaptation of the N170 as well. We hypothesized that if the N170 reflects solely the processing of the CONF_1st_ of a face, then the adaptation effect on the N170 should be similar for the normal and stretched adaptor conditions. Alternatively, if the extraction of CONF_2nd_ is also reflected in the N170, then changing the aspect ratio of the adaptor face should decrease the N170 adaptation effect, that is, a smaller amplitude reduction or no amplitude reduction at all is expected when compared to normally proportioned adapters.

## MATERIALS AND METHODS

### PARTICIPANTS

Twelve naive, healthy volunteers (8 females) with normal or corrected-to-normal vision served as subjects (mean age: 21.55 ± 2.42 years) and gave written informed consent. We conform to the protocols approved by the Ethical Committee of the Budapest University of Technology and Economics.

### STIMULI

Face stimuli (gray-scale full-front images, mean luminance = 1.17 cd/m^2^, 3-3 young males and females) were identical to those of Kovacs et al. (2006), having no obvious gender-specific features and were fit behind an oval mask (6°× 5.9°). Female-male pairs were entered into a landmark-based morphing algorithm (Winmorph 3.01). Ten faces, ranging from 100% female to 100% male in 10% steps, were created (leaving out the 50/50% level) and were used as test stimuli. Additional typical female (NORM_F_) and male (NORM_M_) faces were chosen as adapters (luminance = 1.1 cd/m^2^). These images were vertically stretched (STR_F_ and STR_M_) by 110% and horizontally compressed by 37% and were used as adapters as well. The Fourier phase randomized version (Nasanen, 1999) of a normal face was created and served as an adapter in the control (CTRL) condition. This image lacked any shape information while it preserved the amplitude spectrum of the original image. The inclusion of this stimulus condition was necessary for the ERP analysis in order to assess the putative, category-level N170 adaptation effect; that is, the amplitude reduction in response to face repetition when compared to a condition in which the face is preceded by a non-face stimulus (Kovacs et al., 2006, 2007; Kloth et al., 2010). Thus, five adapter conditions (CTRL, NORM_F_, NORM_M_, STR_F_, STR_M_) were used in total. To control for low-level adaptation, and since previous studies suggested that the N170 is, to a large extent, independent of the size of the stimuli (Jeffreys, 1996), all adapters differed in size from the targets (NORM: 6.8° × 6.3°, STR: 6.8° × 2.4°) and the position of the test stimulus varied randomly within a 1° range along the horizontal and vertical dimensions in each trial.

### PROCEDURE

**FIGURE 1:**
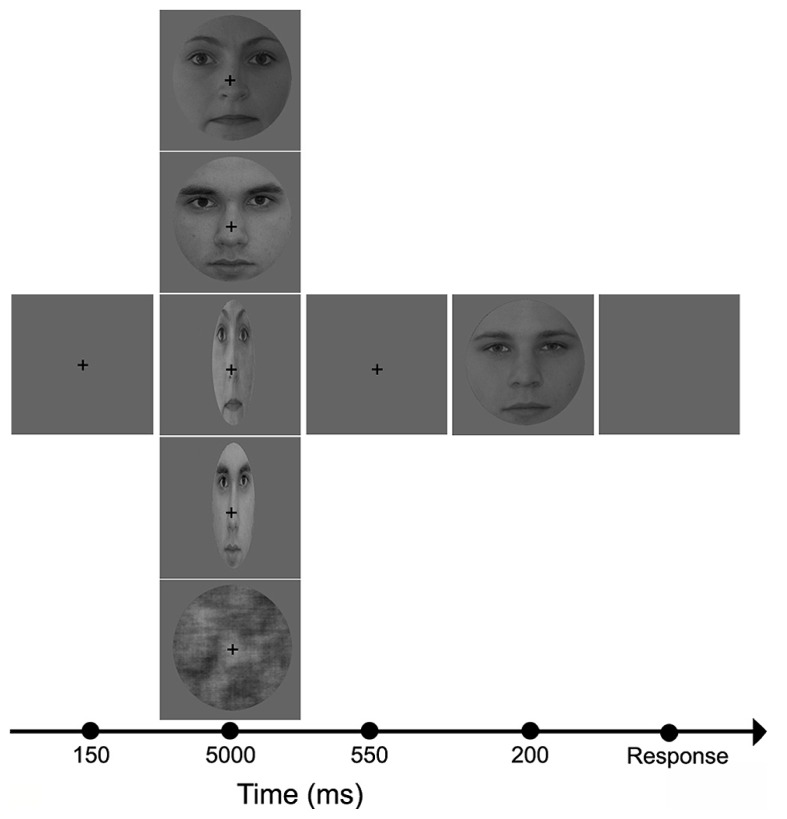
**Experimental protocol and adaptor images.** In the beginning of each trial, a fixation cross was presented in the center of the screen for 150 ms, followed by one of the five adaptor images (from top to bottom: NORM_F_, NORM_M_, STR_F_, STR_M_, and CTRL) which was visible for 5000 ms. This was followed by the presentation of a blank screen for 550 ms, and then the test face was displayed for 200 ms.

Stimuli were presented centrally (21″ monitor, resolution = 1024 × 768, 60 Hz vertical refresh rate; viewing distance = 72 cm) on a uniform gray background (luminance = 1.3 cd/m^2^). The five adaptor conditions were given in separate blocks (pseudo-randomized order). All software was written in MATLAB 6.5 (Mathworks Inc.) using Psychtoolbox 2.45. Subjects were tested in a dimly lit room (background luminance <1 cd/m^2^). They were instructed to fixate a central cross and to perform a two-alternative forced choice gender discrimination task on the test faces. Stimuli were presented according to the method of constant stimuli. The adapter was presented for 5000 ms, followed by a 550 ms gap, and then the test face was presented for 200 ms (**Figure 1**). The five adapter conditions (CTRL, NORM_F_, NORM_M_, STR_F_, STR_M_) were presented in separate blocks with short breaks in between. Within a block, each test stimuli was presented 5 times, yielding 150 trials in each block. The total recording time was approximately 90 min.

### ELECTROPHYSIOLOGICAL RECORDINGS

ERPs were recorded via 32 Ag/AgCl electrodes placed according to the 10/20 system (impedances <5 kømega, sampling rate: 1000 Hz, ground: FT9, reference: AFz). EEG was segmented offline [BrainVision Analyser (Brain Products GmbH)] into 1100 ms long trials including a 100 ms prestimulus interval. Trials containing blinks, movements, A/D saturation or EEG baseline drift were rejected. After artifact rejection 92% of the trials remained available for further analysis. ERPs were averaged separately for each subject, condition and channel. Averages were then digitally filtered (0.5–25 Hz) with a zero phase shift digital filter and were re-referenced to average.

### DATA ANALYSIS

Behavioral data was modeled by the Weibull psychometric function (Psignifit; Wichmann and Hill, 2001). A two-way repeated measures analysis of variance (ANOVA) was conducted with adapter configuration (2 – NORM, STR), adapter gender (2 – F, M) and morph-level (10) as within-subject factors on the participants’ female-male decisions. As we were interested in comparing the aftereffects in case of normal and stretched adapters, and our control stimulus was neither matched to the configuration, nor to the gender of the adaptor faces, we excluded this condition from the statistical analysis. To compare the magnitude of adaptation directly in the NORM and STR conditions, we first calculated the magnitude of the aftereffect by subtracting the percentage of trials endorsed as female obtained during the female adapted conditions from that of the male adapted condition, separately for NORM and STR. Next, the magnitude of aftereffect was subjected to a two-way within-subject ANOVA with configuration (2) and morph-level (10) as factors.

Analyses of the ERP waveforms included the amplitude and latency of three major components: (1) P100 (measured at O1, O2), defined as a main positive deflection around 110 ms and (2) the N170 [P7/P8, P9/P10, PO7/PO8, PO9/PO10; (Eimer, 2000a; Rossion et al., 2000)] and (3) P200 (O1/O2, P5/P6, PO3/PO4, PO7/PO8). After averaging, the individual peak amplitudes were measured for each subject and condition in the time windows of 70-130 ms (P100), 140-210 ms (N170) and 215-320 ms (P200). Latencies were measured at the peak amplitudes. Categorical adaptation effects were determined by comparing the ERP responses found in NORM and STR to those in CTRL. To obtain a sufficient number of trials, data was collapsed across the female and male adaptor conditions as well as across the 10 different target morph-levels (Kovacs et al., 2006; Zimmer and Kovacs, 2011). Amplitude and latency values were entered into a three-way repeated measures ANOVA with adapter type (3, CTRL and NORM or STR), hemisphere (2) and electrode (N170: 4, P200: 4) as within-subject factors. P100 amplitude and latency values were analyzed using a two-way repeated measures ANOVA with adapter type (3) and hemisphere (2) as within-subject factors. All analyses involved Greenhouse-Geisser adjusted degrees of freedom to correct for non-sphericity. *Post hoc* comparisons were made using Bonferroni tests.

## RESULTS

### BEHAVIORAL RESULTS

**FIGURE 2:**
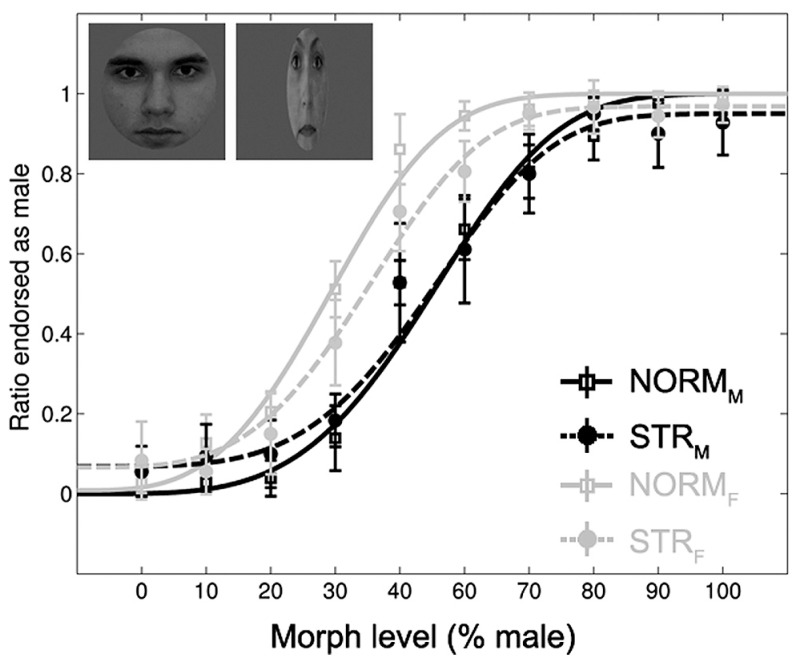
**Mean ratio of stimuli endorsed as male as a function of gender morph level (% male).** Black lines: male, gray lines: female adapters. Continuous lines: adapters with normal proportions (NORM). Dashed lines: stretched adapters (STR). Insets illustrate examples of the adaptor stimuli (NORM_M_ and STR_F_). Data are modeled by a Weibull psychometric function.

Subjects could solve the gender-discrimination task (**Figure 2**), as suggested by the significant main effect of morph-level [*F*(1.98,21.79) = 396.65, *p* < 0.0001, $\eta_{p}^{2}$ = 0.97]. The comparison of female and male adaptor conditions confirmed previous findings in the sense that adaptation to a face with a given gender biases perception towards the opposite gender (Kovacs et al., 2006; Kloth et al., 2010). This is expressed by the fact that significantly more faces were judged as female after being adapted to a male face and vice versa [main effect of adapter gender: *F*(1,11) = 139.17, *p* < 0.0001, $\eta_{p}^{2}$ = 0.93]. In addition, the aftereffect was larger for intermediate than for less ambiguous morph-levels [adapter gender × morph-level interaction: *F*(9,99) = 11.7, *p* < 0.0001, $\eta_{p}^{2}$ = 0.52]. This effect was independent of the adapter configuration as the three-way interaction was not significant [*F*(3.1,34.13) = 1.47, *p* = 0.24, $\eta_{p}^{2}$ = 0.12]. The main effect of configuration tended to show a stronger aftereffect for NORM when compared to STR [*F*(1,11) = 4.43, *p* = 0.059, $\eta_{p}^{2}$ = 0.29] and it showed a significant interaction with adapter gender [*F*(1,11) = 5.54, *p* < 0.05, $\eta_{p}^{2}$ = 0.33]. *Post hoc* tests confirmed the presence of aftereffects in case of NORM and STR adaptors as well; significantly more faces were judged as female following adaptation to either normal (*p* < 0.0001) or stretched male faces (*p* < 0.01) when compared to their female counterparts. No other main effects or interactions were significant.

The direct comparison of the magnitude of aftereffect (see Materials and Methods) for the two configuration conditions showed that the aftereffect is significantly larger for NORM adapters when compared to STR [main effect of configuration: *F*(1,11) = 5.54, *p* = 0.038, $\eta_{p}^{2}$ = 0.33]. The aftereffect was larger for the ambiguous faces when compared to less ambiguous ones [main effect of morph-level: *F*(9,99) = 11.72, *p* < 0.0001, $\eta_{p}^{2}$ = 0.51]. Altogether, these results suggest that adapting to a stretched face is able to bias the perception of a subsequent ambiguous face, but to a lesser degree than a normal, normally proportioned adapter does.

### EVENT-RELATED POTENTIAL RESULTS

**FIGURE 3:**
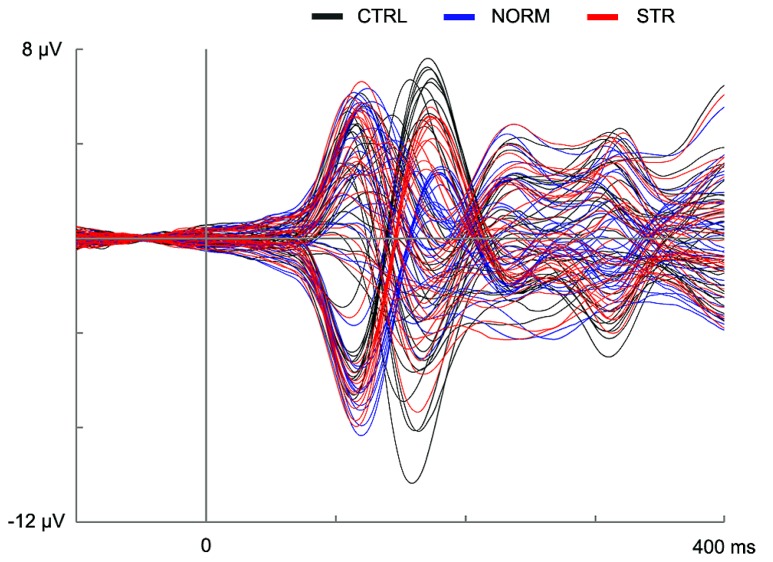
**Event-related potential waveforms for all channels in the CTRL (black lines), NORM (blue lines), and STR (red lines) conditions.** The vertical line at time point 0 marks the onset of the test stimulus.

**FIGURE 4:**
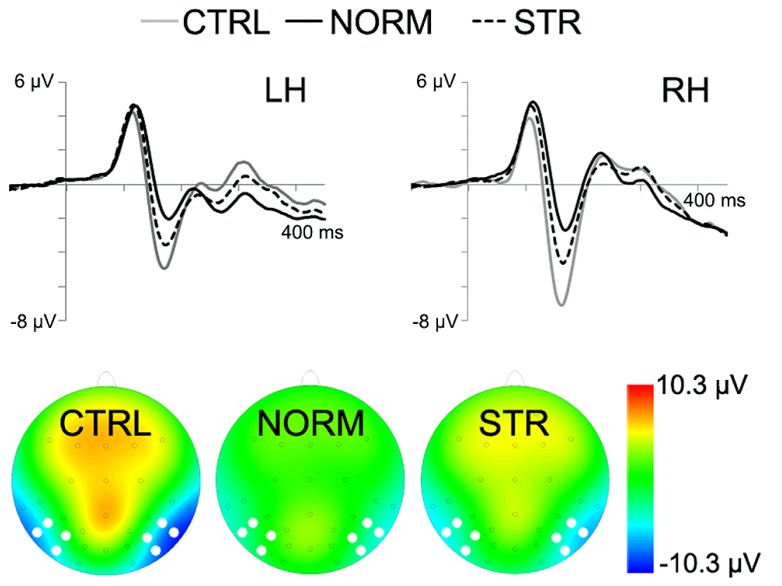
**Grand average ERPs for the CTRL (gray line), NORM (black continuous line) and STR (black dashed line).** The bottom of the figure depicts the topographical maps of the activity with white dots marking the electrode locations, used for N170 analysis (10 ms time-window centered on the peak). Negativity is indicated by blue.

**FIGURE 5:**
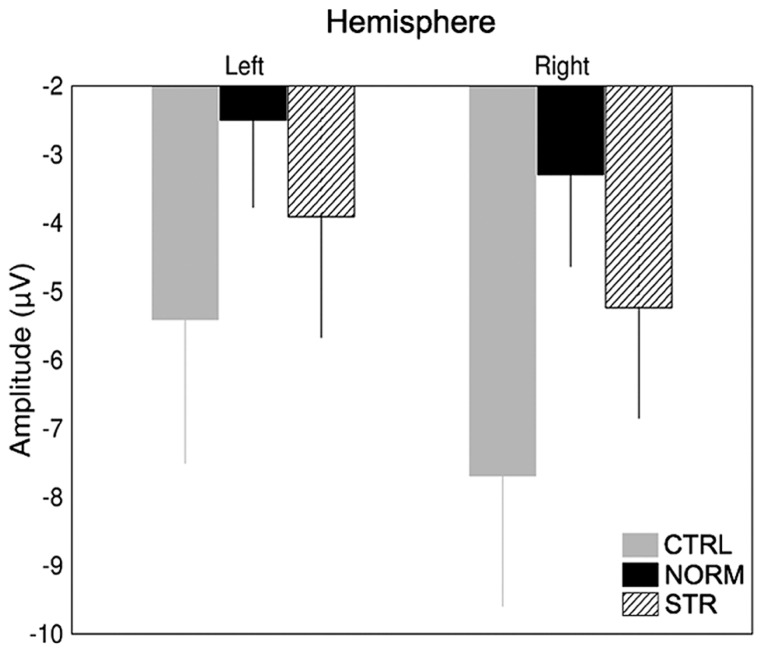
**Average (±SE) amplitude of the N170 component for the two hemispheres and CTRL (gray), NORM (black), and STR (striped) conditions separately**.

The early component peaks P1, N170, and P200 were observable at their typical latencies in the event-related potential following the onset of the test faces (**Figure 3**). The N170 was strongly affected by the type of adaptor image (**Figure 4**) in the sense that both NORM and STR led to lower amplitudes than CTRL [**Figure 5**; main effect of adaptation: *F*(2,22) = 49.44, *p* < 0.0001, $\eta_{p}^{2}$ = 0.82]. This adaptation effect was smaller over the left when compared to the right hemisphere [interaction of hemisphere and adapter condition: *F*(2,22) = 12.6, *p* < 0.001, $\eta_{p}^{2}$ = 0.53] and somewhat larger for more superior (P7, P8, PO7, PO8) when compared to more inferior electrodes [P9, P10, PO9, PO10; electrode × adapter interaction: *F*(1.92,21.07) = 6.3, *p* < 0.01, $\eta_{p}^{2}$ = 0.37]. STR led to lower N170 amplitudes than CTRL (*post hoc* test: *p* < 0.0001 for both hemispheres), reflecting categorical adaptation effects, in spite the changes in CONF_2nd_. However, STR led to significantly higher N170 amplitudes than NORM (*p* < 0.001 for both hemispheres), suggesting that the alterations of CONF_2nd_ modulate the adaptation processes as well.

A significant main effect of adapter condition was observed [*F*(2,22) = 9.93, *p* < 0.01, $\eta_{p}^{2}$ = 0.47] due to the N170 latencies being significantly longer after being adapted to NORM when compared to CTRL (*p* < 0.001). In addition, the latencies were significantly shorter over the right when compared to the left hemisphere [main effect of hemisphere: *F*(1,11) = 17.17, *p* < 0.01, $\eta_{p}^{2}$ = 0.61] and over P9/P10 when compared to the electrodes P7/P8 (*p* < 0.01) and PO7/PO8 [*p* < 0.01; main effect of electrode: *F*(2.13,23.42) = 8.11, *p* < 0.01, $\eta_{p}^{2}$ = 0.42]. Altogether these results suggest that the early and generic structural steps of face processing, reflected in the N170, are sensitive to both the first and second-order configuration changes of the stimuli.

The amplitude of the P200 was significantly higher over the right when compared to the left hemisphere [main effect of hemisphere; *F*(1,11) = 5.02, *p* < 0.05, $\eta_{p}^{2}$ = 0.31]. A significant main effect of electrode [*F*(2.14,23.5) = 4.64, *p* < 0.05, $\eta_{p}^{2}$ = 0.3] was also observed due to the P200 being higher in amplitude over the PO3/PO4 when compared to the P5/P6 electrodes (*p* < 0.01). In addition, a significant adapter condition × hemisphere interaction [*F*(2,22) = 4.86, *p* < 0.05, $\eta_{p}^{2}$ = 0.30] was observed. Post-hoc comparisons, however, failed to show any consistent adaptation effects over either hemisphere. For the latency of the P200, only a significant main effect of electrode was observed [*F*(3,33) = 5.11, *p* < 0.05, $\eta_{p}^{2}$ = 0.32] due to the P200 being longer in latency over the P5/P6 when compared to the O1/O1 (*p* < 0.05) and PO7/PO8 electrodes (*p* < 0.05). Finally, regarding the latency of the P100 we observed a significant main effect of adapter condition [*F*(2,22) = 10.79, *p* < 0.001, $\eta_{p}^{2}$ = 0.5]. Post hoc tests revealed that the P100 peaked later after being adapted to NORM when compared to CTRL (*p* < 0.05) and STR (*p* < 0.001). No other main effects and interactions regarding P100 latencies or amplitudes were significant.

## DISCUSSION

In the present study we tested the effect of CONF_2nd_ on gender-specific aftereffects by vertically stretching the adaptor images. We found contrastive biases in gender perception after adaptation to normal and vertically stretched faces. This finding corroborates the results of previous experiments demonstrating that gender-ambiguous faces are perceived as more masculine after prolonged exposure to a female face and vice versa (Webster et al., 2004; Kovacs et al., 2006; Kloth et al., 2010; Zhao et al., 2011). However, the strength of the aftereffect was smaller for stretched than for unstretched adapters, which suggests that the aftereffects are sensitive to the CONF_2nd_ of the adapters. The pattern of our results implies that gender-specific aftereffects rise partially from processes sensitive to the CONF_2nd_ of faces. This is surprising, given the facts that (1) stretching of a face leaves face-recognition performance unaffected (Hole et al., 2002; Bindemann et al., 2008) and (2) aftereffects are suggested to have greater transfer across transformations preserving identity (Yamashita et al., 2005). Our results challenge this theory (see Tillman and Webster, 2012 for similar conclusions) and suggest that changes of CONF_2nd_ affect gender-specific aftereffects, in spite of their identity-preserving nature. Previous studies have emphasized the role of features in the perception of face gender, since face parts such as the eyebrows, eyes or mouth convey sufficient information for gender discrimination even when they are presented in isolation (Brown and Perrett, 1993; Yamaguchi et al., 1995). Nevertheless, there is evidence for the contribution of relational information to the perception of face gender as well. For example, changes in eyebrow-eyelid distance has been shown to affect gender classification performance (Campbell et al., 1999). Thus, it is possible that stretching faces in the present study altered such relational cues and hence affected the masculinity/femininity of the adaptor faces, which resulted in the decrease of gender-specific aftereffects.

It is important to note that the distortions applied in our study changed substantially the second-order relations of the face; however, they affected the shape of local features as well. The importance of the second-order relations in face discrimination is supported by the observation that differences in the metric distances between facial features play a significant role in perceiving two faces as same or different as well (Rotshtein et al., 2007). Further studies [e.g., by applying the so-called “Jane stimuli” (see Mondloch et al., 2002)] are necessary to investigate the relative contribution of facial features and second-order relations on the N170 adaptation effects.

So far, very few studies have tested the effect of face configuration on N/M170 and these experiments could convincingly show its sensitivity to the CONF_1st_ (Bentin et al., 1996; Eimer, 2000a; Rossion et al., 2000; Gao et al., 2013). Prior results regarding CONF_2nd_ led to unequivocal results with studies emphasizing either the relative insensitivity (Halit et al., 2000; Bindemann et al., 2008; Mercure et al., 2008) or sensitivity (Scott and Nelson, 2006; Zimmer and Kovacs, 2011) of N170 to CONF_2nd_. The current results show category-specific adaptation effects for STR in the form of lower N170 amplitudes when compared to CTRL, but this adaptation effect was smaller than the one observed for NORM. This suggests that the generic, category-specific face-processing steps, reflected in these comparisons of the N170 (Kloth et al., 2010), mirror both the first and second-order properties of stimuli.

Previous studies that failed to demonstrate sensitivity to CONF_2nd_ in the N170 time window typically compared the overall neural response (i.e., the amplitude and latency of the N170) to intact and configurally altered face stimuli (Halit et al., 2000; Mercure et al., 2008). Assessing the effect of stimulus repetition on neural responses, on the other hand, offers a more sensitive method to disentangle the nature of neural representations in a specific brain area or time window. This approach proved to be effective in functional imaging research with the presumption that repetition reduces neural activity only if the subsequently presented stimuli activate the same neural population. This allows for the identification of separate subpopulations of neurons selective for a particular stimulus attribute whose responses cannot be discerned when measuring the overall neural activity (Grill-Spector et al., 2006; Krekelberg et al., 2006). In this respect, our results complement previous findings demonstrating that face-selective areas of the human occipito-temporal cortex show less adaptation to repeated faces when they differ in their second-order relations (Rhodes et al., 2009). To conclude, it is possible that the modulation of the neuronal responses by adaptation is more sensitive to the relatively small changes of CONF_2nd_ stimulus manipulations when compared to the absolute electrophysiological response (for the comparison of stimulus selectivity of neural response and adaptation see Sawamura et al., 2006), explaining why previous studies did not find the N170 to be sensitive to CONF_2nd_.

Previous studies have shown that face gender aftereffects are accompanied by the reduction of the BOLD signal in the fusiform face area and the occipital face area (Kovacs et al., 2008; Nagy et al., 2012). On the basis of these results, it is possible that the aftereffects observed in the present study reflect the adaptation of these face-selective cortical areas; however, this claim should be investigated with functional imaging methods.

Evidence is surprisingly scarce regarding the physiological mechanisms underlying face adaptation aftereffects. It has been shown that cholinergic mechanisms play a role in the face repetition effects observed in the fusiform gyrus (Thiel et al., 2002). In the somatosensory domain, the contribution of glutamatergic neural systems to perceptual adaptation has been demonstrated (Folger et al., 2008). Thus, while certainly speculative, it is possible that cholinergic and glutamatergic neurotransmitter pathways play a role in the face adaptation effects we observed. Further studies could investigate this possibility by means of specific neuro-pharmacological testing.

## CONCLUSION

The present results demonstrate that facial aftereffects evoked by adaptation to normal or vertically stretched faces show sensitivity to second-order relations of facial features. In accordance with the behavioral results, adaptation effects on the N170 ERP component were present, but were smaller in magnitude, after being adapted to stretched faces, suggesting the sensitivity of N170 to second-order relations manipulated by linear distortion.

## AUTHOR CONTRIBUTIONS

Designed the experiment: Stefan R. Schweinberger, Gyula Kovács, Márta Zimmer; data acquisition: Kornél Németh, Pál Vakli; data analyses: Kornél Németh, Pál Vakli, Gyula Kovács; interpretation of the data: Kornél Németh, Pál Vakli, Stefan R. Schweinberger, Gyula Kovács; provided materials: Kornél Németh, Pál Vakli, Márta Zimmer, Gyula Kovács; wrote the article: Kornél Németh, Pál Vakli, Márta Zimmer, Stefan R. Schweinberger, Gyula Kovács; proofed/revised the article: Stefan R. Schweinberger, Kornél Németh, Márta Zimmer, Gyula Kovács.

## Conflict of Interest Statement

The authors declare that the research was conducted in the absence of any commercial or financial relationships that could be construed as a potential conflict of interest.
